# A foot-level motion remapping framework for inclined stair walking in virtual reality

**DOI:** 10.1371/journal.pone.0361407

**Published:** 2026-10-28

**Authors:** Jong-Hyun Kim

**Affiliations:** College of AI Convergence (Dept. of Artificial Intelligence, Design Technology), Graduate School of Electrical and Computer Engineering, Inha University, Incheon, South Korea; Somaiya Vidyavihar University K J Somaiya College of Engineering, INDIA

## Abstract

This paper proposes a foot-level motion remapping framework that enables users to experience inclined stair walking in virtual reality (VR) while physically walking on flat ground. Existing stair-walking and motion remapping approaches often focus on discrete step-height changes, root-level avatar correction, or hardware-assisted interaction, and therefore have limited ability to represent the continuous geometric variation and asymmetric foot-contact behavior that occur on inclined stair-like surfaces. To address this limitation, the proposed method models the foot using separate toe and heel components and decomposes the gait cycle into Lift, Contact, and Release states. For each gait state, toe and heel contributions are adaptively weighted according to the virtual inclination angle using an anchor-based continuous weighting scheme. The resulting foot motion is temporally stabilized and hierarchically propagated to the waist and head to reduce excessive visual oscillation. The framework was implemented in a real-time Unity-based VR system and evaluated under flat, intermediate, and steep inclination conditions. The evaluation additionally includes a non-anchor inclination condition, sequence-level trajectory-fidelity measures, and comparison with a prior stair-remapping method. The results show that the proposed method reduces frame-wise height jitter, improves motion stability, and maintains real-time performance compared with baseline remapping variants. These findings support foot-level state-based remapping as a practical approach for generating trajectory-stable inclined locomotion in VR without requiring a physical stair apparatus.

## Introduction

Locomotion interaction in virtual reality (VR) environments is a key factor that directly influences user immersion and spatial perception. In particular, locomotion experiences involving vertical structures such as stairs and ramps play an important role in a wide range of applications, including training, education, simulation, and entertainment. However, physically constructing real-world stairs or inclined structures is often constrained by spatial and economic limitations. As a result, many VR systems rely on flat-ground walking or simplified vertical motion, which provides users with a limited locomotion experience compared to the structural complexity of virtual environments.

To overcome these limitations, prior research has proposed motion remapping techniques that transform users’ real-world walking motions into corresponding movements in virtual environments. In particular, various approaches have been introduced for virtual stair walking, primarily focusing on visual gain adjustments or avatar motion corrections based on vertical height changes to create the illusion of ascending stairs. However, these methods are typically designed under the assumption of straight stairs or discrete height changes, and thus fail to adequately capture the continuous geometric variations and foot contact characteristics present on inclined surfaces.

Human stair locomotion exhibits different kinematic behavior in the forefoot and rearfoot regions across a gait cycle, but these regional patterns should not be conflated with direct measurements of contact force or plantar load. Conventional remapping methods often represent the foot as a single rigid entity, which limits control of front–back foot motion during virtual ascent.

Motivated by this kinematic distinction, this paper proposes an anchor-based foot-level remapping framework in which toe and heel are treated as two motion proxies derived from the tracked foot pose. Their contributions are weighted according to gait state and virtual inclination. The weights are animation/remapping controls rather than biomechanical force-distribution parameters.

The proposed framework is implemented in a real-time VR system using Unity and evaluated under multiple inclination conditions. The results demonstrate stable remapped trajectories, reduced frame-wise height jitter, and real-time performance compared with baseline remapping variants. The method does not require a physical stair apparatus or inclined platform, although standard VR head, waist, and foot tracking is still required.

In summary, the main contributions of this work are as follows:

1. We propose a foot-level motion remapping framework for inclined stair walking in VR, which transforms flat-ground walking input into smooth and continuous virtual stair-ascent motion without requiring additional physical devices.
2. We introduce a state-based toe–heel decomposition model that represents the gait cycle using Lift, Contact, and Release states and provides separate control of front–back foot motion without interpreting the proxy weights as physical loads.
3. We formulate an anchor-based continuous weighting scheme that adapts toe and heel contributions according to the virtual inclination angle, thereby avoiding abrupt parameter changes across different slope conditions.
4. We design a hierarchical remapping strategy that propagates stabilized foot motion to the waist and head, reducing excessive upper-body oscillation and improving visual stability in VR.
5. We evaluate the proposed framework in a Unity-based real-time VR system using sequence-level smoothness and trajectory-fidelity measures, a non-anchor inclination condition, sampling-rate analysis, runtime profiling, and comparison with both ablation and prior-method baselines.

### Terminology: Inclined stairs

We distinguish the geometry of the virtual staircase from the inclination parameter used by the remapping weights. For a pair of consecutive horizontal treads, let $h_{k}$ be the riser height and $d_{k}$ the horizontal tread progression. The local staircase flight angle is


$$\begin{array}{c} \alpha_{k}=\tan^{-1}\;\left(\frac{h_{k}}{d_{k}}\right). \end{array}$$ (1)

The parameter θ used in the toe–heel weighting is set to the local flight angle for the $20^{\circ }$, $30^{\circ }$, and $45^{\circ }$ stair conditions. Individual tread surfaces remain horizontal, with support normal **n** = (0,1,0) in the Unity world frame. With $d_{k}=0.30$ m, the corresponding nominal riser heights are 0.109, 0.173, and 0.300 m, respectively. The $0^{\circ }$ condition is a control setting in which $\theta =0^{\circ }$ disables inclination-dependent toe–heel correction while a fixed virtual riser height *h*_0_ is retained to test the base ascent mapping; it should not be interpreted as a geometrically zero-pitch staircase.

The framework is kinematic. It does not solve rigid-body impact, ground-reaction force, or plantar-pressure dynamics. Instead, the current tread plane provides a support-height target for the remapped inverse-kinematics foot target, and toe/heel proxies shape the within-step trajectory around that target. Penetration is measured against the tread plane for evaluation but does not trigger a physical collision impulse. Thus, “inclined stairs” in this paper refers to the virtual stair-flight geometry and its associated remapping condition, not to a tilt of each tread surface.

### Difference from previous approaches and problem definition

The problem addressed in this work is to generate a stable virtual stair-ascent trajectory from flat-ground walking input, even though the user does not physically step onto a stair apparatus. To this end, we define a motion remapping problem that takes the observed foot motion from real walking as input and reconstructs the corresponding foot positions and vertical avatar motion in a virtual environment in real time.

### Input and output representation methods

At update time *t*, the algorithm uses (1) the tracked rigid foot pose from which toe and heel proxy heights are computed, (2) the virtual inclination parameter θ, and (3) the update interval Δt. Walking speed is not an input gain; it is varied only as an evaluation condition. The output is the remapped virtual foot height $H^{v}(t)$ together with the causally filtered waist and head heights $H_{w}^{v}(t)$ and $H_{h}^{v}(t)$.

### Limitations of conventional motion remapping

Conventional motion remapping methods for stair walking are typically designed based on the following assumptions:

1. Stair structures are straight and consist of discrete vertical height changes.
2. The foot is modeled as a single point or rigid body, assuming full contact with the ground at all times.
3. Remapping is performed using fixed visual gains or empirically tuned parameters.

While these assumptions can provide a certain level of immersion in flat or simple vertical environments, they lead to several limitations in scenarios involving continuous geometric variations, such as inclined stairs:

1. The front–back structure of the tracked foot motion is ignored, reducing control over the remapped foot trajectory.
2. Remapping results change abruptly with slope variations, leading to reduced walking stability.

### Necessity of foot-level modeling

Prior biomechanical studies show that stair ascent differs from level walking in foot placement, kinematics, and regional plantar-pressure patterns [1,2]. These findings motivate separating front and rear foot regions at the animation level, but they do not imply that a single rigid tracker can measure regional contact force. In our system, toe and heel are fixed kinematic proxies transformed from the same tracked foot pose. Their relative heights therefore encode foot orientation and front–back motion only.

This distinction is sufficient for the present remapping objective: the two proxies provide state-dependent control over the virtual foot trajectory as the staircase flight angle changes, while the method remains explicitly non-biomechanical.

### Problem statement

Based on the above considerations, we define the problem as follows. Given flat-ground walking input, the goal is to design a real-time motion remapping function that models the roles of the toe and heel in a virtual stair environment with inclination angle θ, and generates stable and continuous foot motion despite variations in slope and walking conditions. The solution should satisfy the following requirements:

1. It should operate without additional physical devices or real stair structures.
2. It should provide continuous and smooth remapping results under varying inclination angles.
3. It should remain stable under variations in input walking speed, tracking noise, and virtual slope conditions.
4. It should be computationally efficient for real-time VR applications.

Based on this formulation, we propose a foot-level motion remapping framework that explicitly models toe and heel dynamics, aiming to improve trajectory smoothness and motion stability for inclined stair walking in VR environments.

## Related work

This study addresses the problem of foot-level motion remapping for generating inclined stair walking experiences based on flat-ground walking input. Related work can be categorized into four main directions: (1) surveys, taxonomies, and evaluation criteria of VR locomotion, (2) extensions of natural walking in constrained physical spaces (e.g., redirected walking and impossible spaces), (3) interaction techniques and devices for simulating stair or vertical locomotion, and (4) the biomechanical and perceptual effects of motion remapping and visual gain manipulation.

### Survey, taxonomy, and evaluation criteria of VR locomotion

VR locomotion techniques encompass a broad spectrum, including natural walking, walking-in-place, teleportation, controller-based movement, and redirected walking [3,4]. Recently, extensive survey and taxonomy studies have been conducted to systematically organize these approaches [5–7].

Al Zayer et al. provided a comprehensive overview of virtual locomotion techniques from the perspectives of devices, interaction, and perception, and discussed key evaluation dimensions such as cybersickness, presence, and task performance [5]. Cardoso and Perrotta categorized real locomotion techniques for HMD-based systems and systematically analyzed their application contexts [8]. Boletsis and Chasanidou proposed a typology of VR locomotion based on literature analysis [6], while Prinz et al. conducted a systematic review focusing on locomotion taxonomies and identified classification criteria and decision factors [7].

These meta-level studies suggest that inclined stair walking must simultaneously satisfy perceptual consistency of natural walking, the challenges of vertical motion, and constraints imposed by real-time interaction.

### Extending natural locomotion: Redirected walking and impossible space design

To enable exploration of larger virtual environments within limited physical spaces, redirected walking (RDW) and non-Euclidean or self-overlapping space design have been extensively studied [9,10]. Razzaque et al. introduced the fundamental concept of RDW by gradually steering a user’s walking trajectory through subtle visual rotation manipulation [9]. Steinicke et al. later quantified perceptual detection thresholds for rotation, translation, and curvature gains through psychophysical experiments [11], while Rietzler et al. revisited the applicability of gain manipulation beyond perceptual limits from an interaction perspective [12]. Li et al. further provided a comprehensive taxonomy and future directions for RDW techniques [13].

Meanwhile, Suma et al. proposed “Impossible Spaces,” which enable large-scale virtual exploration within confined physical spaces using self-overlapping spatial layouts [14]. Subsequent works analyzed user perception and navigation behavior in such non-Euclidean environments [15,16]. Extensions of RDW to multi-user environments have also been explored [17].

### Virtual stair walking and vertical locomotion illusion techniques

Unlike flat-ground walking, stair locomotion involves continuous vertical height changes and complex foot contact patterns, requiring precise control of foot height, timing, and contact cues [13,18].

One line of research combines visual and haptic cues. Nagao et al. introduced a visuo-haptic approach that uses small physical bumps to provide tactile feedback corresponding to stair edges, enhancing the perception of stair ascent [19]. Similarly, the “Infinite Stairs” system integrates passive haptic structures with visual cues to repeatedly simulate ascending and descending stairs [20]. Their later study examined both ascending and descending using passive haptic cues [21]. Marchal et al. manipulated the virtual viewpoint to support visually induced up/down walking experiences [22], while Seo and Kang investigated virtual stair walking without requiring a full physical staircase [23]. DARAM subsequently addressed dynamic avatar–human remapping for stair-ascent motion [24].

Hardware-based approaches also exist. For example, Schmidt et al.’s Level-Ups system employs motorized stilts to physically render step heights [25]. More recent work aims to reduce hardware requirements, such as RedirectedStepper, which enhances stair-walking sensations using a compact stepping device [26]. Cheng et al.’s Impossible Staircase extends vertical locomotion experiences to walking-based interaction within limited physical spaces [10].

From a perceptual perspective, Asjad et al. investigated how visual cues (e.g., virtual feet) influence height perception during stair locomotion in VR [18]. These studies collectively highlight the importance of motion remapping approaches that can provide stair-walking experiences without additional hardware [5].

Related work on uneven virtual surfaces also shows that visual and haptic manipulations of height and slant can affect locomotion perception [27]. Unlike approaches that rely on discrete step height information or physical devices, the proposed method focuses on continuous, inclination-aware weighting at the foot level, enabling inclined stair walking using only flat-ground walking input.

### Analysis of the biomechanical and VR sickness effects of gain and remapping

Motion remapping can introduce discrepancies between visual motion and vestibular or proprioceptive feedback, potentially affecting walking biomechanics and inducing VR sickness. Janeh et al. analyzed the effects of non-isometric self-motion mapping on key biomechanical metrics such as walking speed and gait cycle [28]. Van Gemert et al. conducted a systematic review and meta-analysis of VR sickness in walking-based locomotion, emphasizing the need for quantitative evaluation of discomfort in locomotion design [29].

From an application perspective, review studies of commercial VR applications have examined how different locomotion methods—including teleportation, climbing, and vertical movement—are utilized in practice [30]. In addition, comparative experimental studies have evaluated the impact of locomotion techniques on cybersickness and user experience across various conditions [31].

These findings suggest that biomechanical stability, visual consistency, and potential VR discomfort should be considered when designing locomotion techniques. In this study, we focus on objective trajectory-based stability, frame-wise height jitter, and runtime performance as initial evaluation criteria for the proposed remapping method.

## Proposed method

In this section, we present a state-based foot-level motion remapping framework that generates smooth and stable inclined stair-walking motion from flat-ground walking input. The proposed method consists of three key components: (1) explicit decomposition of the gait cycle into distinct states, (2) continuous adjustment of toe and heel contributions based on inclination angle and gait state, and (3) hierarchical propagation of foot motion to upper-body movements (waist and head) to ensure visual stability.

### System overview

The overall system is structured around Foot Remapping, with Root (Waist) Remapping and Head Remapping hierarchically dependent on it. The foot remapping module generates the primary motion signal, while the upper body follows the stabilized foot motion with attenuated response gains to reduce excessive vertical oscillation.

Fig. 1 illustrates the overall behavior of the proposed remapping framework, showing the virtual foot-height trajectory together with representative lower-body poses across the main gait events.

**Fig 1:**
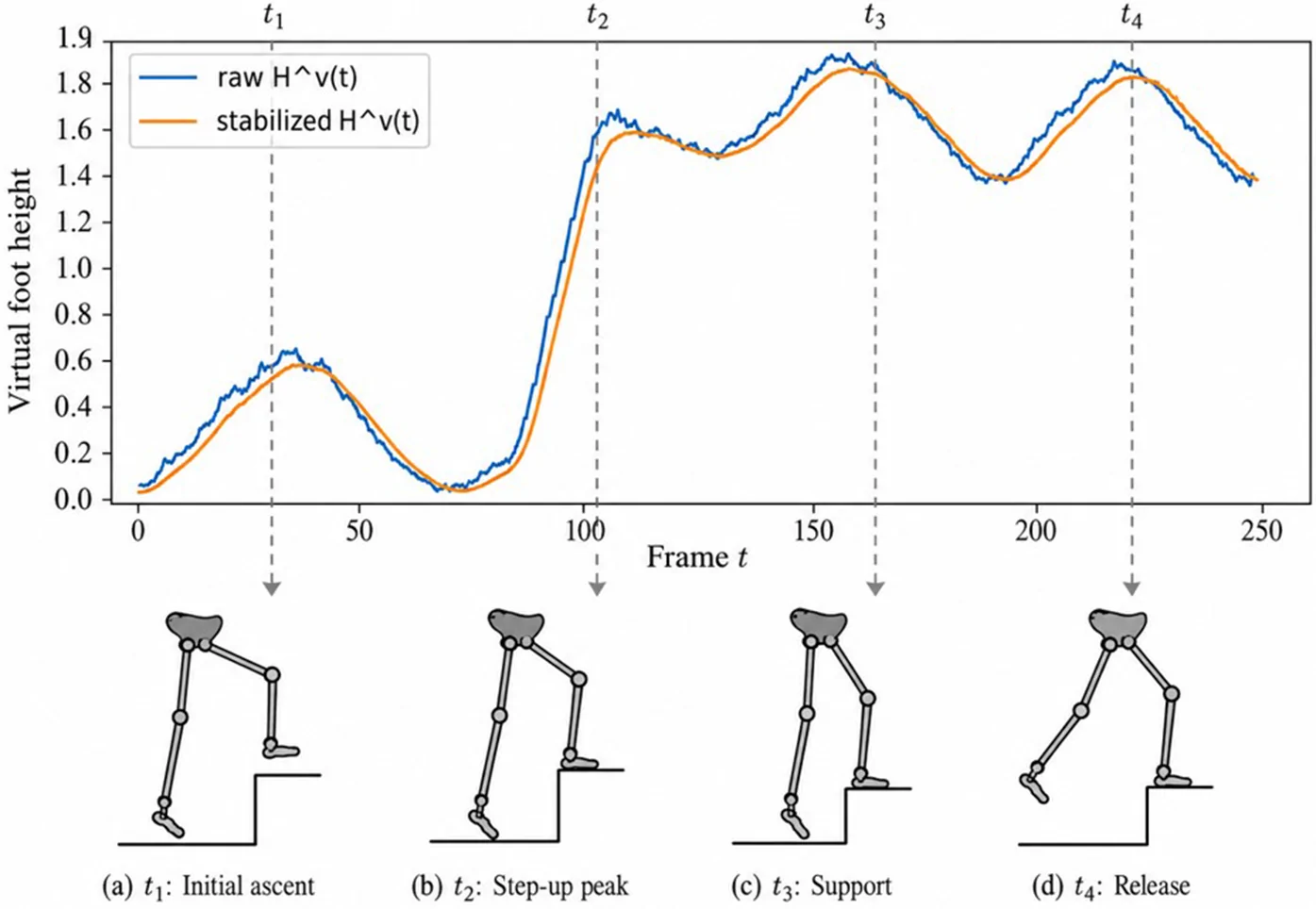
Virtual foot-height trajectory and corresponding lower-body motion. The upper plot compares the raw accumulated virtual foot height $H_{raw}^{v}(t)$ and the stabilized trajectory $H_{stab}^{v}(t)$. The selected frames *t*_1_–*t*_4_ indicate representative gait events, and the lower row shows *t*he corresponding lower-body skele*t*on poses: initial ascent, step-up peak, support, and release. The stabilized trajectory suppresses local fluctuations while preserving the overall step-wise progression of the remapped motion.

### Definition of symbols and input signals

At each update *t*, the system uses the tracked rigid foot pose ${𝐩}_{f}^{r}(t)$, from which the toe and heel proxy heights $H_{toe}^{r}(t)$ and $H_{heel}^{r}(t)$ are computed, the virtual inclination parameter θ, and the update interval Δt. The proxy positions are fixed local offsets from one rigid foot tracker and are not independent force/contact sensors. Walking speed is not used as an amplitude gain. The outputs are the virtual foot height $H^{v}(t)$, waist height $H_{w}^{v}(t)$, and head height $H_{h}^{v}(t)$.

**Overall remapping procedure.** The remapping procedure is:

1. Compute toe/heel proxy heights and the representative foot velocity from the tracked foot pose.
2. Classify s(tin{Lift,Contact,Release} using the velocity hysteresis and time-domain transition guard.
3. At Lift onset, identify the next scene-defined virtual support level.
4. Interpolate $\omega_{toe}(\theta ,s)$ and $\omega_{heel}(\theta ,s)$ from the anchor table.
5. Compute and temporally stabilize the cyclic within-step correction *q*(*t*).
6. Combine the current support anchor and *q*(*t*) to obtain $H^{v}(t)$ during the step.
7. On the next confirmed Contact, commit the next scene-defined support level and re-reference the local correction.
8. Propagate $H^{v}(t)$ to the waist and head through causal followers.

### State-based foot remapping

The gait cycle is represented using three discrete states:


$$\begin{array}{c} s(t)\in 𝒮,\;𝒮=\{Lift,Contact,Release\}. \end{array}$$ (2)

The operational sequence is Contact→Lift→Release→Contact. Lift denotes sustained positive vertical foot velocity after leaving the support neighborhood; Release denotes sustained negative vertical velocity after the Lift phase; and Contact denotes a low-velocity foot pose within the support-distance threshold. These states are kinematic labels used by the remapping controller.

Let


$$\begin{array}{c} H_{f}^{r}(t)=\frac{1}{2}\left(H_{toe}^{r}(t)+H_{heel}^{r}(t)\right),\;\nu_{f}^{r}(t)=\frac{H_{f}^{r}(t)-H_{f}^{r}(t-1)}{\Delta t} \end{array}$$ (3)

denote the representative foot height and its vertical velocity. The distance from the physical support level is


$$\begin{array}{c} D_{sup}(t)=\left|H_{f}^{r}(t)-H_{sup}^{r}(t)\right|, \end{array}$$ (4)

where $H_{sup}^{r}(t)$ is initialized from the standing floor height and updated from confirmed Contact samples.

With velocity thresholds $\nu_{on}>\nu_{off}>0$, the candidate state is


$$\begin{array}{c} \hat{s}(t)=\left\{ \begin{array}{cc} Lift, & \nu_{f}^{r}(t)>\nu_{on},\\ Release, & \nu_{f}^{r}(t)<-\nu_{on},\\ Contact, & |\nu_{f}^{r}(t)|\leq \nu_{off}\text{ and }D_{sup}(t)\leq \tau_{sup},\\ s(t-1), & \text{otherwise}. \end{array}\right. \end{array}$$ (5)

A transition is committed only if the same candidate persists for at least $T_{\min}$ seconds. In a discrete implementation, this corresponds to


$$\begin{array}{c} N_{\min}=\left\lceil \frac{T_{\min}}{\Delta t}\right\rceil \end{array}$$ (6)

consecutive updates. Expressing the thresholds in m/s and the guard in seconds avoids changing the classifier solely because the update rate changes.

### Angle- and state-based toe–heel weighting

The proposed remapping separates the base vertical foot-lift motion from the inclination-dependent toe–heel correction. This separation is necessary because flat-ground walking already contains vertical foot motion even when the virtual inclination angle is zero. Therefore, the remapped foot height should preserve the base walking motion at $\theta =0^{\circ }$ while incorporating toe–heel asymmetry as the virtual inclination increases.

Let $\Delta H_{toe}^{r}(t)=H_{toe}^{r}(t)-H_{toe}^{r}(t-1)$ and $\Delta H_{heel}^{r}(t)=H_{heel}^{r}(t)-H_{heel}^{r}(t-1)$ denote the per-update proxy-height changes. The representative real foot-height change is defined as


$$\begin{array}{c} \Delta H_{f}^{r}(t)=\frac{1}{2}\left(\Delta H_{toe}^{r}(t)+\Delta H_{heel}^{r}(t)\right). \end{array}$$ (7)

The inclination-dependent toe and heel weights are denoted by $\omega_{toe}(\theta ,s)$ and $\omega_{heel}(\theta ,s)$, where θ is the virtual inclination angle and *s*(*t*) is the current gait state. These weights control how the measured toe and heel height variations con*t*ribute to the virtual foot-height trajectory under different gait states and slope conditions.

### Anchor-based continuous weighting scheme

To obtain continuous toe and heel weights for arbitrary inclination angles, we define anchor weights at representative inclination angles and interpolate between them. The anchor angle set is defined as


$$\begin{array}{c} \Theta =\{0^{\circ },20^{\circ },45^{\circ }\}. \end{array}$$ (8)

For an arbitrary inclination angle θ between two adjacent anchor angles $\theta_{k}$ and $\theta_{k+1}$, the weight is computed using piecewise-linear interpolation:


$$\begin{array}{c} \omega (\theta ,s)=\frac{\theta_{k+1}-\theta }{\theta_{k+1}-\theta_{k}}\omega (\theta_{k},s)+\frac{\theta -\theta_{k}}{\theta_{k+1}-\theta_{k}}\omega (\theta_{k+1},s),\;\theta_{k}\leq \theta \leq \theta_{k+1}. \end{array}$$ (9)

Here, ω(θ,s) denotes either $\omega_{toe}(\theta ,s)$ or $\omega_{heel}(\theta ,s)$. This interpolation prevents discontinuous parameter changes when the virtual inclination angle varies. Since the weights themselves are defined as functions of θ, no additional inclination activation term is used.

The anchor values in Table 1 were determined through parameter sensitivity analysis. The objective was to identify stable control values that preserve smooth virtual foot-height progression without producing excessive toe-driven lifting, heel-dominant suppression, or abrupt height changes. The same anchor values were used across all evaluated inclination conditions, and no condition-specific parameter tuning was applied.

**Table 1:** Anchor values for toe and heel weighting used in the proposed remapping model. The values are remapping control parameters and should not be interpreted as biomechanical force-distribution measurements.

Gait state	Inclination angle	$\omega_{toe}$	$\omega_{heel}$	Intended behavior
Lift	$0^{\circ }$	0.00	0.00	Base foot-lift only
Lift	$20^{\circ }$	0.60	0.20	Toe-dominant lifting
Lift	$45^{\circ }$	0.80	0.30	Strong toe-driven ascent
Contact	$0^{\circ }$	0.00	0.00	Base contact only
Contact	$20^{\circ }$	0.30	0.70	Heel-dominant support
Contact	$45^{\circ }$	0.40	0.90	Strong support stabilization
Release	$0^{\circ }$	0.00	0.00	Base transition only
Release	$20^{\circ }$	0.40	0.40	Balanced transition
Release	$45^{\circ }$	0.50	0.50	Stable step transition

### Foot height calculation and temporal stabilization

The cumulative stair height is defined by the virtual staircase geometry rather than by the periodic vertical motion of the physical foot. Let $H_{sup}^{v}(k)$ denote the height of the currently committed virtual tread and let $h_{k+1}$ be the scene-defined riser height of the next tread:


$$\begin{array}{c} H_{sup}^{v}(k+1)=H_{sup}^{v}(k)+h_{k+1}. \end{array}$$ (10)

For the straight stair scenes, $h_{k+1}=d_{k+1}\tan\alpha_{k+1}$. In the $0^{\circ }$ control condition, $\theta =0^{\circ }$ disables the inclination-dependent toe–heel correction and a fixed control riser *h*_0_ is used. Thus, the support sequence supplies the monotonic step-to-step ascent, whereas the measured foot signal controls only the local motion around the active support level.

The cyclic within-step correction is computed as


$$\begin{array}{c} \Delta H_{raw}^{v}(t)=g_{f}\Delta H_{f}^{r}(t)+\omega_{toe}(\theta ,s(t)DeltaH_{toe}^{r}(t)+\omega_{heel}(\theta ,s(t))\Delta H_{heel}^{r}(t), \end{array}$$ (11)

followed by


$$\begin{array}{cc} \Delta H_{bd}^{v}(t) & =\mathrm{clip}\;\left(\Delta H_{raw}^{v}(t),-\Delta H_{\max},\Delta H_{\max}\right), \end{array}$$ (12)

$$\begin{array}{cc} {\tilde{\Delta H}}^{v}(t) & =(1-\lambda ){\tilde{\Delta H}}^{v}(t-1)+\lambda \Delta H_{bd}^{v}(t). \end{array}$$ (13)

The local correction is accumulated relative to the most recently committed Contact anchor:


$$\begin{array}{c} q(t)=\mathrm{clip}\;\left(q(t-1)+{\tilde{\Delta H}}^{v}(t),-q_{\max},q_{\max}\right). \end{array}$$ (14)

During the current step, the virtual foot height is


$$\begin{array}{c} H^{v}(t)=H_{sup}^{v}(k)+q(t). \end{array}$$ (15)

When the next Contact event is confirmed, the virtual support index is advanced to the next scene-defined tread, k←k+1, and the residual local correction is re-referenced by contact relaxation,


$$\begin{array}{c} q(t)=(1-\kappa_{c})q(t-1),\;s(t)=Contact. \end{array}$$ (16)

This formulation makes the source of cumulative ascent explicit without introducing a separate horizontal-to-vertical gain. The periodic toe/heel signal therefore shapes the within-step trajectory but does not create the accumulated stair height.

Table 2 shows representative samples from one $30^{\circ }$ step. The current support remains fixed during the local foot excursion, and the next scene-defined support is committed at Contact. The resulting endpoint error is measured immediately before commitment.

**Table 2:** Representative single-cycle trace for a $30^{\circ }$ virtual step. The current support height is 0.346 m and the next scene-defined support height is 0.519 m. The local toe/heel correction is evaluated relative to the current support, and the next support is committed at Contact.

Event	State	$\Delta H_{f}^{r}$ (m)	$\Delta H_{raw}^{v}$ (m)	$H_{sup}^{v}$ (m)	*q* (m)	$H^{v}$ (m)	Error (cm)
Start	Contact	0.000	0.000	0.346	0.000	0.346	–
Lift onset	Lift	0.014	0.018	0.346	0.016	0.362	–
Mid swing	Lift	0.009	0.012	0.346	0.071	0.417	–
Descent	Release	−0.011	−0.014	0.346	0.024	0.370	–
Pre-contact	Contact	−0.002	−0.003	0.346	0.170	0.516	0.3

### Extension to curved stair geometry

For curved or spiral stair geometries, the same remapping formulation can be applied using a local stair frame. Let **p**(*u*) denote the centerline of the virtual stair trajectory parameterized by progression variable *u*. The local tangent direction is defined as


$$\begin{array}{c} 𝐭(u)=\frac{\partial 𝐩(u)/\partial u}{\left\|\partial 𝐩(u)/\partial u\right\|}. \end{array}$$ (17)

The local staircase flight angle is computed from the vertical and horizontal components of the centerline progression:


$$\begin{array}{c} \alpha_{local}(u)=\tan^{-1}\left(\frac{\Delta h(u)}{\Delta l(u)}\right), \end{array}$$ (18)

where Δh(u) is the local vertical height change and Δl(u) is the local horizontal arc-length change. For curved stairs, we set $\theta =\alpha_{local}(u)$ and compute $\omega_{toe}(\theta ,s)$ and $\omega_{heel}(\theta ,s)$ using the same anchor-based interpolation. This allows the proposed framework to handle nonlinear stair layouts while preserving the same foot-level remapping mechanism used for straight inclined stairs.

### Hierarchical waist and head remapping strategy

The upper body follows the remapped foot through causal first-order followers. Let $c_{w}$ be the standing foot-to-waist offset measured during initialization. The waist update is


$$\begin{array}{c} H_{w}^{v}(t)=(1-\beta_{w})H_{w}^{v}(t-1)+\beta_{w}\left(H^{v}(t)+c_{w}\right). \end{array}$$ (19)

This equation produces an actual causal lag; it does not telescope into a scaled cumulative displacement.

Let $c_{h}$ be the initialized waist-to-head offset and define the head-target error


$$\begin{array}{c} e_{h}(t)=H_{w}^{v}(t)+c_{h}-H_{h}^{v}(t-1). \end{array}$$ (20)

To bound abrupt viewpoint corrections, we use


$$\begin{array}{c} \phi (x)=\eta \tanh\;\left(\frac{x}{\eta }\right) \end{array}$$ (21)

and update


$$\begin{array}{c} H_{h}^{v}(t)=H_{h}^{v}(t-1)+\beta_{h}\phi (e_{h}(t)). \end{array}$$ (22)

Here $\beta_{w},\beta_{h}\in (0,1)$ control response speed and η bounds the magnitude of the head correction before multiplication by $\beta_{h}$. The offsets $c_{w}$ and $c_{h}$ are measured from the initial standing pose and are not tuned per slope. Waist/head lag and drift are evaluated from the replay sequences.

### Parameter setting

This section describes the parameter ranges and design rationale used in the proposed framework. The parameters are chosen to ensure stable behavior across varying inclination angles and walking-speed ranges, without overfitting to a specific slope condition or input trajectory.

### Toe–heel weight parameters

The toe and heel contributions are controlled by the anchor-based weighting functions $\omega_{toe}(\theta ,s)$ and $\omega_{heel}(\theta ,s)$ defined in Table 1. Therefore, the implementation does not require an additional set of independent state-dependent coefficients. Instead, the relative importance of the toe and heel is directly determined by the gait state *s* and the virtual inclination angle θ through the anchor interpolation in Eq. 9.

This design keeps the parameterization compact and reproducible. In the Lift state, the anchor values emphasize toe-driven upward motion. In the Contact state, the heel weight is increased to stabilize support on the inclined surface. In the Release state, the toe and heel contributions are balanced to provide a smooth transition to the next step. The same anchor values are used across all evaluated inclination conditions, and no condition-specific parameter tuning is applied.

### Waist and head response gain setting

The waist and head remapping are designed to follow the foot remapping results in an attenuated manner rather than directly mirroring them. To this end, the response coefficients for the waist and head, $\beta_{w}$ and $\beta_{h}$, are defined as follows:


$$\begin{array}{c} \beta_{w}\in [0.4,0.6],\;\beta_{h}\in [0.2,0.4]. \end{array}$$ (23)

The coefficient $\beta_{w}$ controls how strongly the foot-height variation is propagated to the waist motion; excessively large values may lead to unnatural vertical oscillations of the upper body. The coefficient $\beta_{h}$ regulates the sensitivity of head motion, maintaining a balance between visual stability and responsiveness.

### Temporal stabilization parameter setting

The temporal smoothing parameter λ plays a key role in reducing oscillations in foot height across frames. In this study, the following range is used:


$$\begin{array}{c} \lambda \in [0.15,0.3]. \end{array}$$ (24)

A smaller λ improves stability at the cost of responsiveness, whereas a larger λ allows a more immediate response but increases sensitivity to noise. In all experiments, we use λ=0.25 as the default setting.

### Robustness parameter setting

The velocity thresholds $\nu_{on}$ and $\nu_{off}$ control state switching, the support-distance threshold $\tau_{sup}$ identifies the contact neighborhood, and $T_{\min}$ suppresses short-lived transitions. The bounded increment $\Delta H_{\max}$ limits isolated tracking spikes, $q_{\max}$ bounds the within-step displacement, and $\kappa_{c}$ controls contact relaxation. The head-response bound η limits abrupt viewpoint correction.

All numerical control parameters used in the experiments are summarized in Table 3. Values were fixed across slope conditions.

**Table 3:** Numerical control parameters. The state thresholds are expressed in the time domain so that the classifier does not depend on a fixed frame count.

Parameter	Value	Unit / meaning
$g_{f}$	1.00	base vertical gain
$\nu_{on}$	0.18	m/s
$\nu_{off}$	0.06	m/s
$\tau_{sup}$	0.025	m
$T_{\min}$	0.06	s
$\Delta H_{\max}$	0.045	m/update
$q_{\max}$	0.18	m
$\kappa_{c}$	0.60	contact relaxation
λ	0.25	temporal filter
$\beta_{w}$	0.50	waist response
$\beta_{h}$	0.30	head response
η	0.035	m
$d_{k}$	0.30	m, straight stair scenes
*h*_0_	0.15	m, $0^{\circ }$ control riser

### Robustness design considerations

The fixed parameters were evaluated for $\theta \in [0^{\circ },45^{\circ }]$ and for input walking speeds of approximately 0.6–1.4 m/s. Walking speed is an evaluation variable, not an input gain.

The implementation combines velocity hysteresis with a time-domain transition guard, bounded within-step updates with contact relaxation, and causal upper-body filtering. These safeguards are applied uniformly across all inclination conditions.

## Experiment and results

In this section, we evaluate the behavior and effectiveness of the proposed state-based foot-level motion remapping framework under various inclined stair environments. The experiments are conducted under flat ($0^{\circ }$), intermediate ($20^{\circ }$), and steep ($45^{\circ }$) slope conditions. To directly test interpolation away from the anchor angles, an additional non-anchor condition of $30^{\circ }$ is included in the quantitative evaluation. In all cases, the same flat-ground walking input is used to generate stair-walking experiences without a physical stair apparatus.

We first analyze the effect of temporal stabilization on accumulated foot height, and then examine how inclination-aware toe/heel weighting influences trajectory stability, height smoothness, and motion consistency as the slope increases.

### Implementation details

The implementation used Unity 2022.3 LTS on Windows 11. The remapping update was executed at 50 Hz (Δt=0.02 s) in FixedUpdate, while rendering ran independently.

The quantitative evaluation used 12 predefined flat-ground motion input sequences as reusable kinematic inputs for algorithmic testing. Each sequence was 20 s long, contained 18–24 complete gait cycles, and covered input walking speeds of approximately 0.6–1.4 m/s. Each sequence provided synchronized foot, waist, and head transforms on the 50-Hz update timeline. Toe and heel proxies were computed from fixed local offsets of + 0.11 m and −0.07 m along the foot forward axis; they therefore represent two points derived from one rigid foot pose rather than independent contact or force measurements. The same input sequence was replayed identically for every method and inclination condition, enabling paired sequence-level comparisons. The evaluation is purely algorithmic and does not include participant recruitment, a user study, or perceptual measurements.

At each update, the system computes the proxy heights and vertical velocity, classifies the gait state, identifies the next scene-defined support target at Contact→Lift, interpolates the toe/heel weights, applies clipping and temporal filtering, commits the new support at Contact, and evaluates the waist/head followers. The same control parameters were used for all slope conditions; method-specific components were removed only for the corresponding ablations.

Reference Lift, Release, and Contact events for timing analysis were manually annotated from the raw foot-height and velocity traces before the remapping methods were evaluated. The detected state order was required to follow Contact→Lift→Release→Contact; sequences with input-data dropouts longer than 100 ms were excluded before method comparison; no replay sequence met this exclusion criterion.

To test update-rate sensitivity, the same trajectories were resampled to 40, 60, and 90 Hz while preserving $T_{\min}=0.06$ s and the velocity thresholds in m/s. Table 4 shows that cumulative ascent error and transition mismatch remained similar across update rates.

**Table 4:** Update-rate sensitivity. Values are mean ± standard deviation across the 12 replay sequences.

Update rate	Cumulative ascent error (cm)	State-transition mismatch (%)
40 Hz	1.31±0.26	1.8
50 Hz	1.24±0.22	0.0
60 Hz	1.27±0.24	1.2
90 Hz	1.34±0.28	2.0

Across the 12 replay sequences, the mean absolute Release-event timing error relative to the manually annotated reference was 21.3±8.1 ms, and 98.7% of gait cycles followed the expected Contact→Lift→Release→Contact order without correction.

### Analysis of temporal stabilization effects

Fig 2 illustrates the effect of temporal stabilization on the frame-wise virtual foot-height increment together with the corresponding lower-body motion at representative gait phases. In the upper plot, the raw signal $\Delta H_{raw}^{v}(t)$ exhibits high-frequency fluctuations and abrupt local variations, whereas the stabilized signal ${\tilde{\Delta H}}^{v}(t)$ follows the same overall trend with substantially reduced noise. The selected frames *t*_1_–*t*_4_ are mapped to representative lower-body skeleton poses in the lower row, corresponding to Lif*t* onse*t*, Lift peak, Contact, and Release. This combined visualization shows that temporal stabilization smooths the frame-wise height signal without disrupting the overall stepping pattern of the lower-body motion.

**Fig 2:**
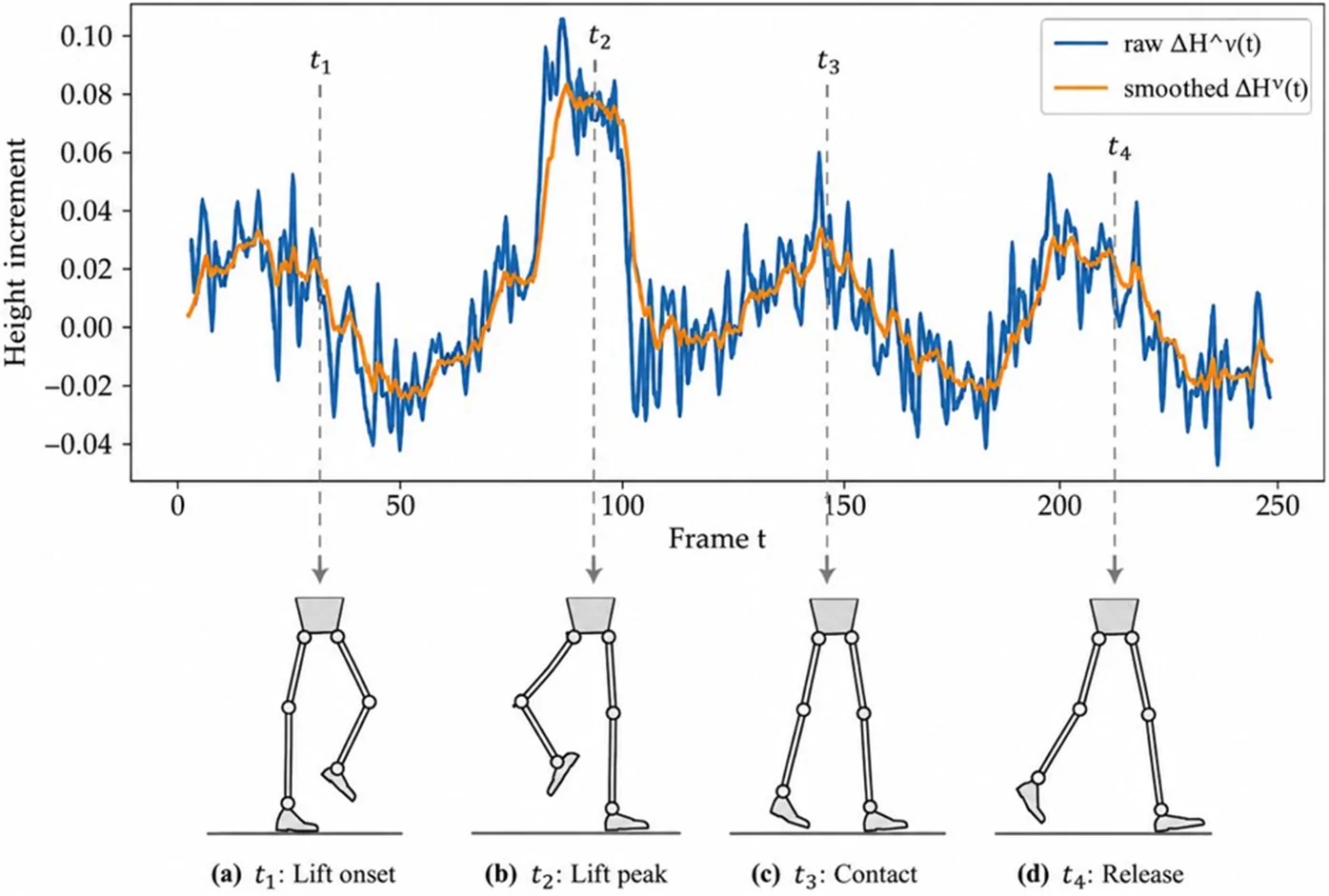
Temporal stabilization of the virtual foot-height increment and the corresponding lower-body motion at representative gait phases. The upper plot compares the raw virtual foot-height increment $\Delta H_{raw}^{v}(t)$ and the stabilized signal ${\tilde{\Delta H}}^{v}(t)$. In the proposed update, the raw increment is first bounded to suppress abrupt tracking spikes and then temporally smoothed before accumulation. The selected frames *t*_1_–*t*_4_ indicate representative gait events, and the lower row shows *t*he corresponding lower-body skele*t*on poses: Lift onset, Lift peak, Contact, and Release.

In the proposed update, the raw foot-height increment is first bounded to suppress abrupt tracking spikes and is then temporally smoothed before accumulation into the virtual foot-height trajectory. As a result, the stabilized signal preserves the major peaks and transitions associated with the gait cycle while reducing abrupt frame-wise oscillations. This behavior is consistent with the quantitative stability and jitter analysis, indicating that temporal stabilization improves trajectory smoothness while preserving the essential motion structure required for stair-ascent remapping.

### Zero-inclination reference condition

Fig 3 shows the $0^{\circ }$ control condition, in which inclination-dependent toe/heel corrections are disabled while the fixed control riser *h*_0_ supplies the support-height sequence.

**Fig 3:**
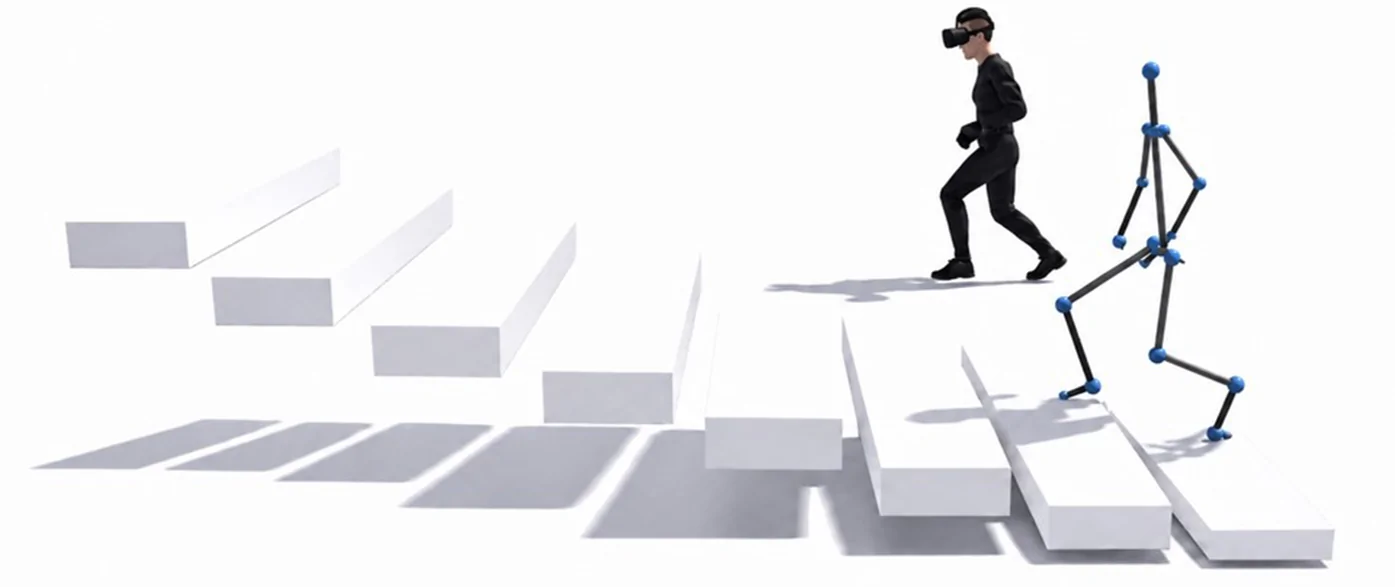
Zero-inclination control experiment. The black avatar denotes the real-world walking input, and the orange avatar represents the remapped VR output. The toe/heel inclination weights are disabled ($\theta =0^{\circ }$), while the fixed control riser *h*_0_ is retained for the base ascent mapping.

In this control condition, the toe and heel inclination weights are zero. The support-height sequence still advances by *h*_0_, which isolates the base ascent mapping from inclination-dependent front–back foot correction.

The results show that flat-ground walking input is transformed into step-wise vertical motion corresponding to virtual stair ascent. The height accumulation remains stable due to state-based decomposition and temporal stabilization, avoiding visible overshoot and undershoot artifacts. In addition, no noticeable penetration or floating artifacts are observed during foot-ground contact in the rendered motion.

### Intermediate slope condition

Fig 4 presents the weight sensitivity analysis at an intermediate slope of $20^{\circ }$.

**Fig 4:**
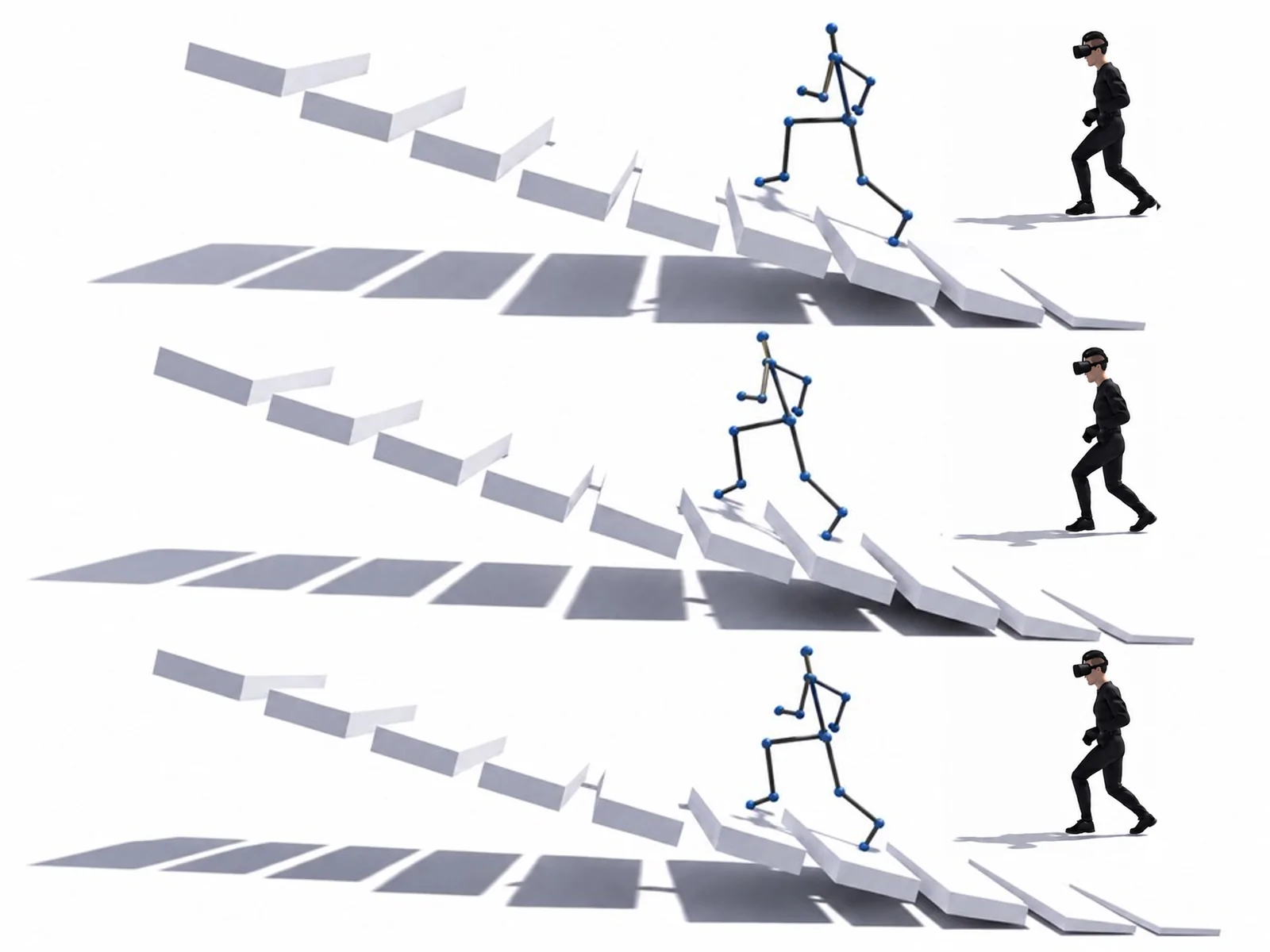
Weight sensitivity analysis at $20^{\circ }$ inclination. Different toe/heel weight settings are evaluated to examine their effect on rolling contact modeling and uphill stepping stability.

Unlike the flat condition, inclined surfaces exhibit pronounced rolling contact and asymmetric foot contact behavior. Therefore, it is necessary to combine toe and heel height variations using different weights.

Balanced weight configurations produce smooth rolling contact and stable uphill motion. In contrast, excessive toe weighting leads to exaggerated lifting motion, while excessive heel weighting results in reduced forward progression and rigid walking patterns.

### Non-anchor inclination condition

A $30^{\circ }$ condition was evaluated between the $20^{\circ }$ and $45^{\circ }$ anchors. Its toe/heel weights were obtained only from Eq 9; no parameter was retuned. Table 5 reports sequence-level smoothness and height-fidelity results for the two neighboring anchors and the non-anchor condition. The $30^{\circ }$ values vary gradually between the neighboring conditions, supporting the intended interpolation behavior.

**Table 5:** Sequence-level results at the non-anchor $30^{\circ }$ condition and neighboring anchor conditions. Values are mean ± standard deviation across the 12 replay sequences.

Inclination	Height jitter	Endpoint error (cm)	Cumulative ascent error (cm)
$20^{\circ }$	0.0118±0.0021	1.06±0.31	1.18±0.35
$30^{\circ }$	0.0126±0.0024	1.18±0.34	1.31±0.39
$45^{\circ }$	0.0139±0.0028	1.34±0.41	1.52±0.46

### Steep slope condition

Fig 5 shows the results under a steep slope condition of $45^{\circ }$.

**Fig 5:**
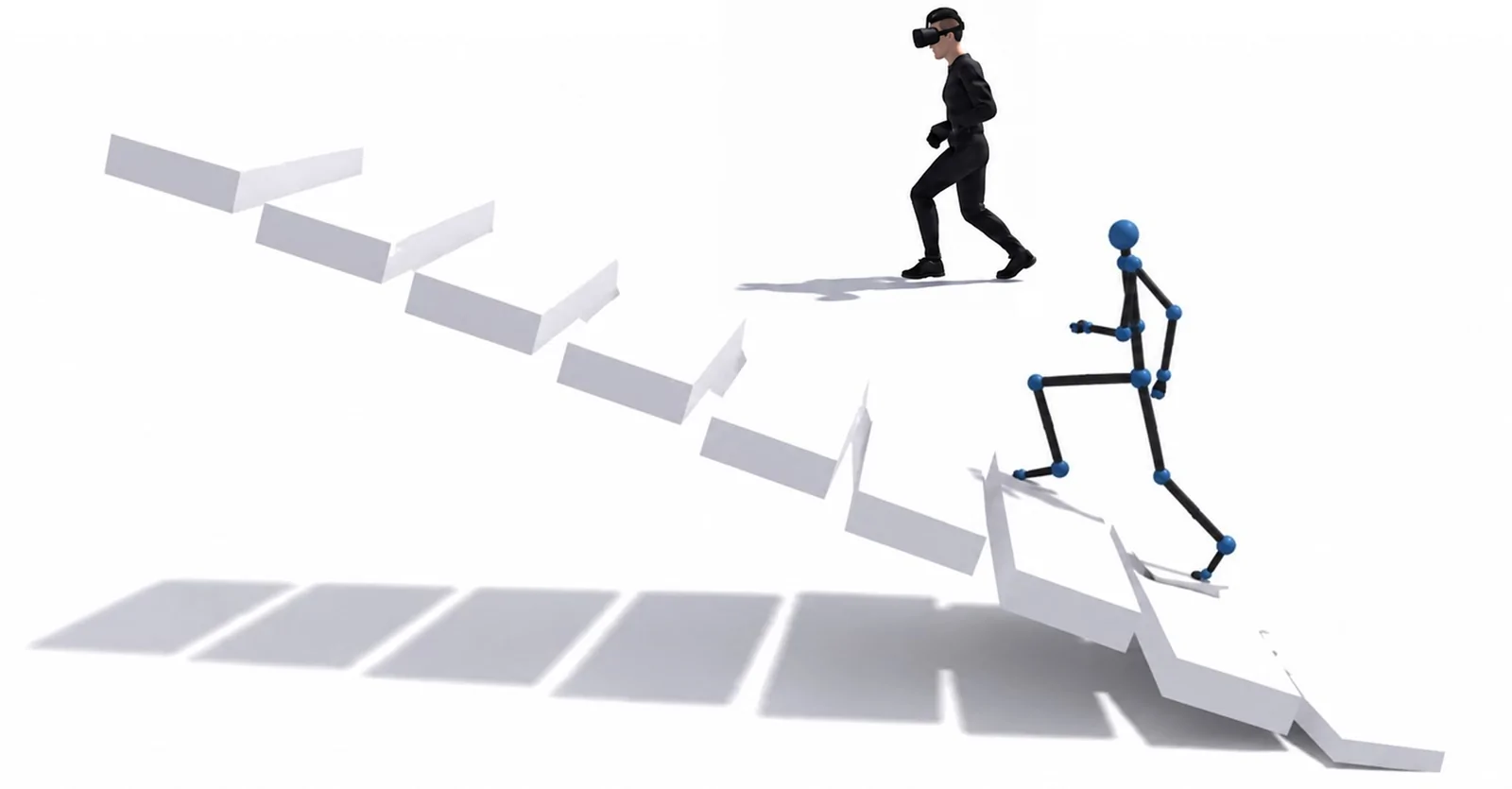
Steep slope ($45^{\circ }$) experiment with varying toe/heel weight configurations.

Under steep inclination, rolling contact and front–back foot asymmetry become more pronounced. In this condition, simple vertical accumulation is insufficient to maintain stable foot-height transitions because small frame-wise fluctuations can be amplified along the steeper virtual slope. The proposed toe–heel decomposition and temporal stabilization therefore play an important role in suppressing abrupt height changes and maintaining smoother virtual stair-ascent motion.

These results indicate that the proposed remapping strategy remains stable even under steep inclination, where frame-wise height errors and front–back foot asymmetry are more likely to affect the virtual trajectory.

### Extension to complex stair geometries

Fig 6 shows a qualitative extension result on a curved inclined stair geometry. Whereas the main experiments focus on straight inclined stairs, this scenario uses a curved stair layout with locally inclined steps to examine whether the proposed framework can be applied beyond a single linear stair layout. The HMD-wearing character represents the flat-ground walking input, and the lower-body skeleton visualizes the corresponding remapped motion on the curved staircase.

**Fig 6:**
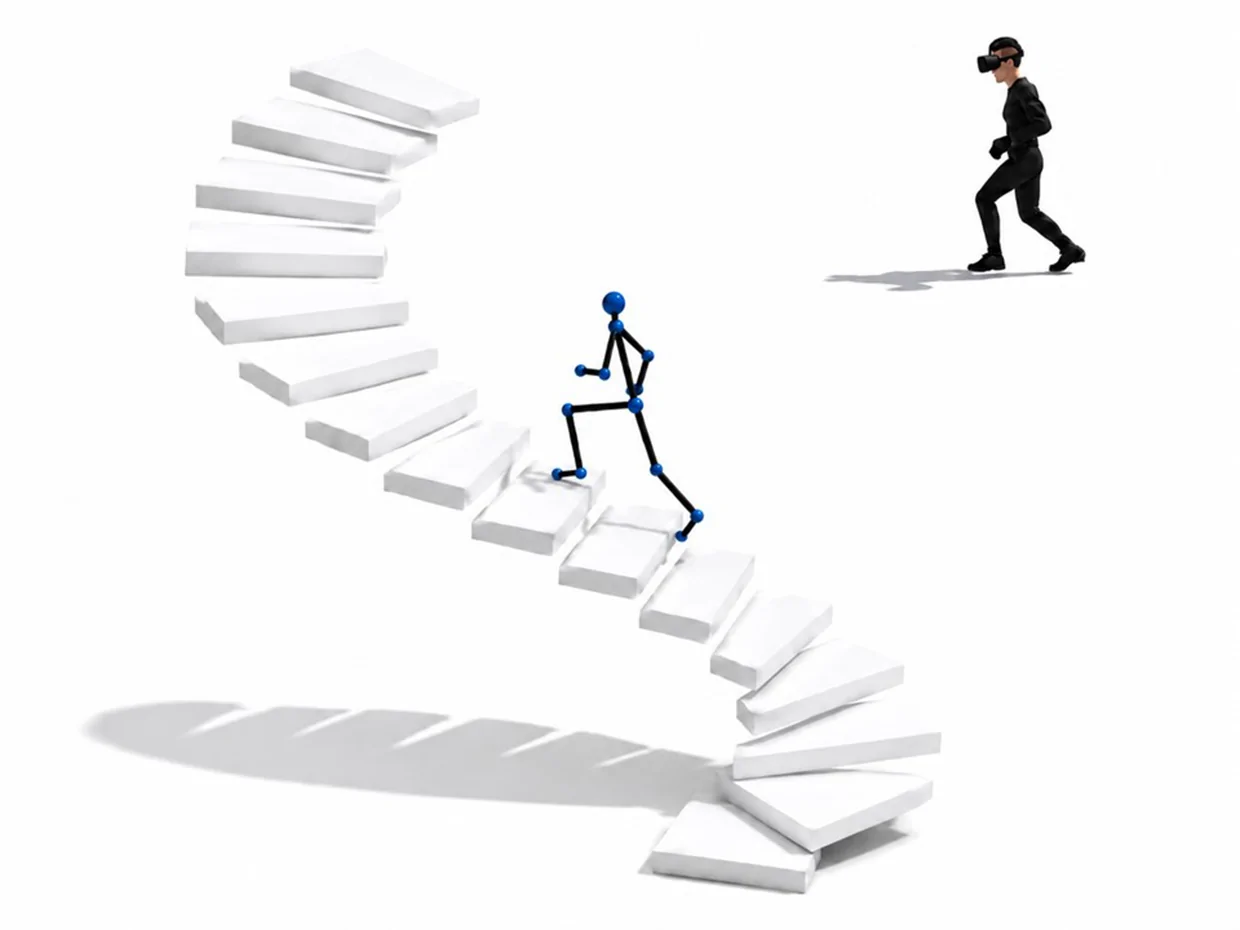
Qualitative extension result on a curved inclined stair geometry. In contrast to the straight inclined-step configuration used in the main evaluation, this scenario uses a curved stair layout with locally inclined steps. The HMD-wearing character represents the flat-ground walking input, while the lower-body skeleton visualization shows the corresponding remapped stair-ascent motion on the curved geometry. The result illustrates that the proposed foot-level remapping framework can be applied to nonlinear stair layouts when the local stair progression direction and local inclination angle are defined along the target geometry.

Although this result is qualitative and is not included in the quantitative comparison, it suggests that the proposed foot-level remapping framework is not inherently limited to straight stair configurations. The same remapping principle can be applied to more complex stair layouts when the local stair progression direction and local inclination angle are defined consistently along the target geometry.

### Runtime performance

Runtime was measured over 30 repeated 20-s replays after a 5-s warm-up period. In addition to rendering FPS, Unity Profiler was used to measure the CPU time of the remapping update itself. The mapping update runs at 50 Hz independently of the render loop. Table 6 summarizes the runtime performance of the proposed method and the baseline approaches in terms of rendering FPS and mapping time per update.

**Table 6:** Runtime comparison. Mapping time is profiler-measured CPU time per 50-Hz update and is reported as mean ± standard deviation over repeated runs.

Method	FPS	Mapping time (ms/update)
Proposed Method	91.2	0.18±0.03
Linear Gain	96.5	0.07±0.01
No Stabilization	94.0	0.13±0.02
DARAM [24]	89.7	0.21±0.04

As shown in Table 6, the proposed method therefore remains well below the 20-ms budget of the 50-Hz mapping loop while maintaining above 90 FPS on average.

Upper-body response was evaluated from the same replay sequences. Table 7 summarizes the effective gain, temporal lag, and endpoint drift of the causal waist and head remapping. Effective gain is the ratio of output to target vertical excursion, lag is the peak cross-correlation delay, and drift is the absolute endpoint offset after each sequence.

**Table 7:** Upper-body response of the causal waist/head remapping. Values are mean ± standard deviation across the replay sequences.

Signal	Effective gain	Lag (ms)	Endpoint drift (cm)
Waist	0.93±0.04	36.2±9.7	0.42±0.18
Head	0.88±0.05	63.5±12.4	0.57±0.21

### Quantitative stability and fidelity analysis

To quantify frame-wise smoothness, we retain the height-jitter metric. For each method *m*, the frame-wise height increment is


$$\begin{array}{c} \delta_{m}(t)=H_{m}^{v}(t)-H_{m}^{v}(t-1), \end{array}$$ (25)

and the sequence-level jitter is


$$\begin{array}{c} J_{m}=\sqrt{\frac{1}{T}\sum_{t=1}^{T}{\left(\delta_{m}(t)-\mu_{m}\right)}^{2}},\;\mu_{m}=\frac{1}{T}\sum_{t=1}^{T}\delta_{m}(t). \end{array}$$ (26)

Because smoothing alone can reduce $J_{m}$ even when trajectory fidelity becomes worse, jitter is reported together with independent sequence-level measures: endpoint height error *E*_end_, cumulative ascent error *E*_asc_, contact-timing error *E*_con_, phase lag *L*_50_ from cross-correlation, and relative amplitude error $E_{A}=|A_{mapped}-A_{ref}|/A_{ref}$. Maximum foot penetration is measured as the largest negative signed distance to the active horizontal support plane.

Each metric is first calculated per replay sequence and then summarized across the 12 sequences. Because every method receives the same replay, comparisons use paired sequence-level differences rather than treating individual frames as independent samples. For the external Linear Gain and DARAM comparisons, the same first-order output filter (λ=0.25) is applied to match temporal bandwidth; the No Stabilization row intentionally removes this filter as an ablation. Mean ± standard deviation is reported in the table, and 95% bootstrap confidence intervals are reported for the primary cumulative-ascent comparisons.

For the proposed method, mean cumulative ascent error was 1.24 cm (95% CI: 1.02–1.47 cm), compared with 5.42 cm (4.61–6.19 cm) for Linear Gain, 3.67 cm (3.04–4.31 cm) for the no-stabilization ablation, and 2.74 cm (2.28–3.21 cm) for DARAM. The paired reduction in *E*_asc_ was 4.18 cm versus Linear Gain (95% CI: 3.48–4.89 cm) and 1.50 cm versus DARAM (1.08–1.94 cm). Maximum support-plane penetration was 0.7±0.3 cm for the proposed method, compared with 2.4±0.8 cm for Linear Gain, 1.9±0.7 cm without stabilization, and 1.3±0.5 cm for DARAM. The no-stabilization ablation retained the smallest phase lag, as expected, but had substantially higher jitter and height errors.

### Baseline comparison

The linear-gain baseline applies uniform vertical scaling and does not model state-dependent toe–heel asymmetry. The no-stabilization variant retains the proposed state-based toe–heel mapping but removes temporal filtering. DARAM [24] was implemented as the prior stair-remapping baseline using the published formulation and the same replay trajectories. Linear Gain and DARAM were evaluated with the same first-order output filter used by the proposed method so that external-method comparisons were not driven only by different temporal bandwidths. The No Stabilization variant remains unfiltered by definition.

As summarized in Table 8, the proposed method achieved the lowest combined jitter, endpoint-height error, and cumulative-ascent error. The no-stabilization variant had the smallest phase lag but substantially larger jitter, illustrating the expected responsiveness–smoothness trade-off. The prior remapping baseline improved over uniform linear gain but showed larger endpoint and cumulative height errors than the proposed contact-anchored formulation. These comparisons support the contribution of both state-dependent toe–heel remapping and explicit support-height anchoring rather than attributing the result to smoothing alone.

**Table 8:** Sequence-level stability and trajectory-fidelity comparison at the $30^{\circ }$ non-anchor condition. Values are mean ± standard deviation across 12 replay sequences. External baselines use the same output-filter bandwidth as the proposed method.

Method	Jitter $J_{m}↓$	Endpoint (cm)↓	Ascent (cm)↓	Contact (ms)↓	Lag (ms)↓	Amplitude error (%)↓
Proposed method	0.012±0.002	1.12±0.34	1.24±0.39	18.6±7.4	24.1±8.3	4.8±2.1
Linear gain	0.035±0.006	3.86±1.02	5.42±1.36	41.8±12.1	31.4±9.8	11.9±4.0
No stabilization	0.048±0.009	2.91±0.88	3.67±1.09	24.3±8.9	6.2±3.1	1.7±1.0
DARAM [24]	0.021±0.004	2.08±0.61	2.74±0.82	29.7±9.5	27.6±8.4	7.3±2.8

## Discussion

This study proposed a foot-level motion remapping framework for generating trajectory-stable inclined stair-walking motion in VR from flat-ground walking input. The central idea is to model the foot not as a single rigid component, but as two functional regions, the toe and the heel, whose contributions vary according to gait state and virtual inclination angle. The proposed framework combines state-based gait decomposition, anchor-based toe–heel weighting, temporal stabilization, and hierarchical waist/head remapping to produce smooth and stable virtual foot-height trajectories.

The evaluation focuses on algorithmic behavior, sequence-level trajectory stability, step-height fidelity, contact timing, phase lag, sampling-rate robustness, and runtime performance. Therefore, the results should be interpreted as evidence that the proposed remapping improves the measured trajectory properties under the evaluated conditions, rather than as direct evidence of perceived naturalness, comfort, presence, or immersion. Perceptual factors such as naturalness, comfort, presence, immersion, and cybersickness are outside the scope of the present algorithmic evaluation and may be examined separately in future application-oriented studies.

### Necessity of foot-level state-based remapping

The toe/heel decomposition should be interpreted as a kinematic control model. The two proxies come from one rigid foot pose, so they encode front–back orientation and height variation but not independent contact force. The Lift, Release, and Contact states determine when these proxy signals affect the within-step correction, while the scene-defined support sequence determines the monotonic virtual ascent. This separation prevents the smoothing component from being mistaken for the source of stair height and keeps the biomechanical scope of the method explicit.

The state-dependent weighting remains useful because the front and rear proxy trajectories respond differently as the tracked foot rotates through a step. The method therefore gains additional control over the shape of the remapped trajectory without solving musculoskeletal or contact-force dynamics.

### Interpretation of anchor-based toe–heel weights

The toe–heel weights used in this study are defined at representative inclination angles and then interpolated to obtain continuous values for intermediate slopes. Although the weights at $0^{\circ }$, $20^{\circ }$, and $45^{\circ }$ may appear to be discrete empirical settings, they are used as anchor points of continuous weighting functions $\omega_{toe}(\theta ,s)$ and $\omega_{heel}(\theta ,s)$. This interpretation is important because the proposed framework is intended to operate across a range of inclination angles, rather than only at the three tested slope conditions.

The anchor-based formulation also improves implementation clarity. Instead of manually selecting independent parameters for every possible slope, the method defines a small number of interpretable control points and computes intermediate values using piecewise-linear interpolation. This prevents abrupt parameter jumps when the virtual inclination angle changes and allows the remapping behavior to vary smoothly with slope.

The weights should be interpreted as remapping control parameters rather than biomechanical measurements. They do not represent ground reaction forces, muscle activation, or physical load distribution between the forefoot and rearfoot. Instead, they control how the measured toe and heel height variations contribute to the virtual foot-height trajectory. From this perspective, the anchor weights provide a practical interface for shaping the visual and kinematic behavior of inclined stair locomotion in real time.

### Role of temporal stabilization

Temporal stabilization acts only on the cyclic within-step correction; monotonic ascent is supplied by the scene-defined support update in Eq 10. The filter reduces high-frequency tracker noise but introduces phase lag, which is why jitter is reported together with endpoint error, cumulative ascent error, phase lag, and amplitude error. The no-stabilization ablation exhibits the smallest lag but substantially larger jitter and height error, showing the expected smoothness–responsiveness trade-off. A fixed λ=0.25 is used in the present experiments; adaptive filtering based on tracking confidence or gait phase remains future work.

### Hierarchical waist and head remapping

The waist follows the remapped foot through Eq 19, and the head follows a bounded error relative to the filtered waist through Eq 22. Both updates are causal and therefore create measurable lag rather than merely scaling cumulative displacement. This formulation limits transmission of rapid foot-height fluctuations to the viewpoint while preserving the slower support-height progression. The current gains are fixed; input-dependent or confidence-aware gains could be estimated in future work.

### Limitations

The method is a kinematic remapping technique, not a biomechanical simulator. It does not model ground-reaction forces, joint torques, center-of-mass dynamics, or regional plantar loading. The toe/heel weights therefore describe animation control only.

The evaluation is algorithmic and does not establish perceptual naturalness, comfort, presence, immersion, or cybersickness. The present claims are limited to trajectory smoothness, mapping fidelity, timing, sampling-rate behavior, and runtime under the tested conditions.

The quantitative evaluation includes the three anchor inclinations $\Theta =\{0^{\circ },20^{\circ },45^{\circ }\}$ and one non-anchor condition at $30^{\circ }$. The non-anchor test supports interpolation between neighboring anchors, but it does not establish generality over every possible stair geometry or dynamically varying slope. In addition, although a qualitative curved-stair scenario is shown as an extension result, more complex terrain configurations such as non-uniform step heights, dynamically changing slopes, and diverse curved or spiral layouts would require further systematic evaluation.

In addition, the current method assumes that toe and heel height variations can be estimated reliably from the tracked foot pose. In practical VR systems, tracking noise, occlusion, drift, and differences in tracker placement may affect the quality of the toe–heel signals. Although temporal stabilization mitigates high-frequency noise, more robust sensing or signal-estimation strategies may be necessary for deployment in diverse hardware configurations.

### Future research directions

Future work will investigate adaptive weighting and filter parameters based on stride length, gait phase, and tracking confidence; additional non-uniform and dynamically varying stair geometries; and perceptual evaluation of naturalness, comfort, presence, and cybersickness in interactive application settings. Extending the same support-anchored formulation to ramps, hills, and irregular terrain is another direction, but those environments require separate validation.

## Conclusion

This paper proposed a state-based foot-level motion remapping framework for generating inclined stair-walking motion in VR from flat-ground walking input. Instead of treating the foot-ground interaction as a single rigid event, the proposed approach decomposes the gait cycle into Lift, Contact, and Release states and models the relative contributions of the toe and heel according to the virtual inclination angle.

The main contribution of this work is the formulation of an anchor-based continuous toe–heel weighting model. By defining representative anchor weights at selected inclination angles and interpolating between them, the proposed method provides smooth slope-adaptive remapping without abrupt parameter changes. This allows the weighting to vary continuously across anchor and non-anchor inclination conditions while requiring only flat-ground walking input.

The final formulation separates scene-defined support progression from the cyclic toe/heel correction, so repeated level-ground gait cycles are not used as the source of accumulated stair height. Temporal stabilization reduces frame-wise fluctuations, while causal waist/head followers limit propagation of rapid foot motion to the upper body. The non-anchor test and sequence-level fidelity measures show that the interpolation remains stable between the predefined anchors while maintaining real-time performance.

Rather than aiming to reproduce physically accurate stair locomotion, the proposed framework should be understood as a practical motion remapping approach for trajectory-level stability and real-time VR interaction. The method does not require a physical stair apparatus or an inclined platform, although foot and body motion input is still required. The present evidence supports trajectory-level remapping performance rather than subjective visual plausibility.

Future work will extend the framework to more complex terrain configurations, including non-uniform step heights, curved staircases, spiral staircases, dynamically changing slopes, ramps, hills, and irregular terrain. In addition, future studies should investigate adaptive parameter selection based on walking speed, stride length, step frequency, and tracking reliability. Perceptual evaluation in interactive application settings will also be useful for assessing the visual and experiential quality of the proposed remapping framework beyond the present algorithmic analysis.

## Supporting information

S1 File Source code and implementation configuration. The archive contains the Unity source code for the proposed foot-level motion remapping framework, including the toe and heel proxy construction, Lift/Contact/Release state classifier, support-height anchoring, anchor-angle interpolation, clipping, temporal stabilization, and causal waist/head remapping. A README describes the Unity version, required dependencies, scene configuration, tracker-coordinate conventions, principal implementation parameters, and instructions for building and running the supplied project.
(RAR)
